# Carfilzomib demonstrates broad anti-tumor activity in pre-clinical non-small cell and small cell lung cancer models

**DOI:** 10.1186/s13046-014-0111-8

**Published:** 2014-12-31

**Authors:** Amanda F Baker, Neale T Hanke, Barbara J Sands, Liliana Carbajal, Janet L Anderl, Linda L Garland

**Affiliations:** University of Arizona Cancer Center, College of Medicine, Section of Hematology/Oncology, 1515 N Campbell Ave, Tucson, AZ USA; Onyx Pharmaceuticals, Inc., an Amgen subsidiary, South San Francisco, CA USA

**Keywords:** Carfilzomib, Proteasome inhibitor, Lung cancer, Cisplatin

## Abstract

**Background:**

Carfilzomib (CFZ) is a proteasome inhibitor that selectively and irreversibly binds to its target and has been approved in the US for treatment of relapsed and refractory multiple myeloma. Phase 1B studies of CFZ reported signals of clinical activity in solid tumors, including small cell lung cancer (SCLC). The aim of this study was to investigate the activity of CFZ in lung cancer models.

**Methods:**

A diverse panel of human lung cancer cell lines and a SHP77 small cell lung cancer xenograft model were used to investigate the anti-tumor activity of CFZ.

**Results:**

CFZ treatment inhibited both the constitutive proteasome and the immunoproteasome in lung cancer cell lines. CFZ had marked anti-proliferative activity in A549, H1993, H520, H460, and H1299 non-small cell lung cancer (NSCLC) cell lines, with IC_50_ values after 96 hour exposure from <1.0 nM to 36 nM. CFZ had more variable effects in the SHP77 and DMS114 SCLC cell lines, with IC_50_ values at 96 hours from <1 nM to 203 nM. Western blot analysis of CFZ-treated H1993 and SHP77 cells showed cleavage of poly ADP ribose polymerase (PARP) and caspase-3, indicative of apoptosis, and induction of microtubule-associated protein-1 light chain-3B (LC3B), indicative of autophagy. In SHP77 flank xenograft tumors, CFZ monotherapy inhibited tumor growth and prolonged survival, while no additive or synergistic anti-tumor efficacy was observed for CFZ + cisplatin (CDDP).

**Conclusions:**

CFZ demonstrated anti-proliferative activity in lung cancer cell lines *in vitro* and resulted in a significant survival advantage in mice with SHP77 SCLC xenografts, supporting further pre-clinical and clinical investigations of CFZ in NSCLC and SCLC.

**Electronic supplementary material:**

The online version of this article (doi:10.1186/s13046-014-0111-8) contains supplementary material, which is available to authorized users.

## Background

Over the last several decades, proteasome inhibition has been extensively investigated as a selective anti-cancer strategy and validated in clinical trials using first and second generation proteasome inhibitors (PIs) [1]. Inhibition of the proteasome can induce disturbances in signal transduction, apoptosis regulation, cell cycle control, transcriptional regulation, and inflammation [2]. A dominant mechanism of action that contributes to the anti-tumor activity of proteasome inhibition is the down-regulation of proto-oncogenic nuclear factor kappa B (NF-кB) signaling through the blocking of inhibitory factor kappa B (I-кB) degradation; inhibition of NF-кB signaling reduces expression of pro-inflammatory response genes and upregulates several cycle-dependent kinase inhibitors, promoting tumor cell apoptosis [3]. Other mechanisms by which proteasome inhibitors induce tumor cell apoptosis include phosphorylation and cleavage of the anti-apoptotic factor Bcl-2, stabilization of p53, interference with the unfolded protein response leading to endoplasmatic reticulum stress, and activation of TNF-related apoptosis-inducing ligand-induced apoptosis through increased death receptors DR4 and DR5 [4–7].

Inhibition of the proteasome has proven to be an effective therapeutic strategy for multiple myeloma and mantle cell lymphoma [8,9]. There has been interest in proteasome inhibition as a therapeutic strategy in solid tumors, including lung cancer. Bortezomib (BTZ), the first-in-class Food and Drug Administration approved PI, has been investigated in preclinical models and in clinical trials as an anti-cancer therapeutic for lung cancer. While BTZ showed potent *in vitro* activity in a wide range of non-small cell lung cancer (NSCLC) cell lines and demonstrated significant *in vivo* activity [10], clinical trials with BTZ monotherapy and in combination with chemotherapy or targeted agents in chemotherapy-naïve and previously-treated NSCLC patients yielded overall mixed results [11–18]. In the setting of relapsed/refractory small cell lung cancer (SCLC), a clinical trial of BTZ reported limited single-agent activity [19].

Carfilzomib (CFZ) is a selective PI that is approved in the United States for the treatment of relapsed and refractory multiple myeloma (RRMM). CFZ binds irreversibly to its target, resulting in sustained inhibition, which is in contrast to the reversible, boronate-based PIs, such as BTZ and MLN9708 [20–23]. CFZ selectively inhibits the chymotrypsin-like activity of the constitutive proteasome and the immunoproteasome [21,22]. CFZ, unlike BTZ, has minimal off-target effects on non-proteasome, serine proteases including cathepsin A, cathepsin G, chymase, dipeptidyl peptidase II, and HtrA2/Omi, which is thought to underlie its favorable toxicity profile with less neurotoxicity than BTZ [24]. CFZ overcomes BTZ resistance in some preclinical models, suggesting that selective, irreversible PIs without dose-limiting neurotoxicity may lead to more potent antitumor response and an improved tolerability profile compared with reversible PIs [25]. A phase I/II study of CFZ reported a durable partial tumor response in a patient with heavily pretreated SCLC [26]. Additionally, CFZ has shown clinical activity in some BTZ-treated patients [27,28].

While novel targeted therapy has proven effective in a subset of NSCLC patients, mainly never smokers, there are relatively limited therapeutic options after failure of first-line regimens for both NSCLC and SCLC related to intrinsic and acquired mechanisms of resistance to chemotherapy. There continues to be interest in developing novel molecularly targeted therapeutic strategies for lung cancer. Given the potential for improved efficacy and greater tolerability of CFZ, we investigated the anti-tumor activity of CFZ in NSCLC and SCLC cell line models alone and in combination with cis-diammineplatinum (II) dichloride (cisplatin, CDDP). We report that proteasome inhibition with CFZ resulted in potent *in vitro* growth inhibition and induction of apoptosis across a diverse set of lung cancer cell lines and *in vivo* tumor growth inhibition in a SCLC xenograft model. However, the combination of CFZ with CDDP was not additive or synergistic in a number of cell lines and a SCLC xenograft, suggesting that other rational combinations of CFZ with chemotherapy or targeted agents be investigated.

## Methods

### Reagents and antibodies

CFZ, provided by Onyx Pharmaceuticals, Inc., an Amgen subsidiary (South San Francisco, CA), was dissolved in dimethyl sulfoxide (DMSO) (Sigma-Aldrich, St. Louis, MO) at a stock concentration of 10 mM and stored at −20°C. A stock concentration of 3.3 mM CDDP in saline (Teva Pharmaceuticals, Israel) was stored at −20°C. Antibodies against poly ADP ribose polymerase (PARP), cleaved caspase-3, p-glycoprotein (Pgp; MDR1), and B-cell lymphoma 2 (Bcl-2) were purchased from Cell Signaling Technology (Beverly, MA). Antibodies against microtubule-associated protein-1 light chain-3B (LC3B) were obtained from Sigma-Aldrich. Alpha-tubulin antibodies were purchased from Calbiochem (La Jolla, CA). The secondary antibodies, HRP-conjugated goat anti-rabbit and HRP-conjugated goat anti-mouse, were purchased from Jackson ImmunoResearch (West Grove, PA).

### Cell lines

All NSCLC (NCI-H520, A549, NCI-H1993, NCI-H460, and NCI-H1299) and SCLC (SHP77 and DMS114) cell lines were obtained from the American Tissue and Cell Collection (ATCC). These cells represent different pathological subtypes (squamous, adenocarcinoma, carcinoma) with SCLC cells derived from both metastatic lesions (SHP77) and a primary tumor (DMS114). A variety of molecular characteristics are also represented including wild-type p53 (H549, H460), reduced or deleted p53 (H520, H1299), wild-type KRAS (H1299), mutated KRAS (A549, H460), wild-type EGFR (A549), mutated EGFR (H1993, H460), and amplified c-met (H1993). All cells were cultured in RPMI 1640 (Cellgro) with 10% fetal bovine serum (FBS), 1 mM sodium pyruvate, and 10 mM HEPES. SHP77 cells were maintained in a similar manner except that heat-inactivated FBS was used. All cells were grown in 5% CO_2_ at 37°C in a humidified tissue culture incubator. Cells were routinely tested for mycoplasma contamination using a MycoAlert mycoplasma detection kit (Lonza, Rockland, ME) and were found to be negative. To verify cell line authenticity, genomic DNA was extracted (Sigma GIN70-KT), diluted appropriately in TE buffer, and submitted to the University of Arizona Genomics Core (Human Origins Gentoyping Lab) for analysis. Autosomal short tandem repeat typing was conducted across the 13 core STRs in CODIS and referenced against allelic peaks in cell lines of previously confirmed genotype. All cell lines were verified as authentic.

### Proliferation assay

Cells were seeded in 96-well plates and incubated overnight. Cultures were exposed to various concentrations of CFZ or CDDP for the specified treatment intervals. Proliferation of adherent cells was determined by 3-[4,5-dimethylthiazol-2-yl]-2,5-diphenyltetrazolium bromide (MTT) assay. Proliferation of suspension cells was determined by 3-(4,5-dimethylthiazol-2-yl)-5-(3-carboxymethoxyphenyl)-2-(4-sulfophenyl)-2H-tetrazolium (MTS) assay. For adherent cultures, MTT dye (2 mg/ml) was added, and the cells were incubated for an additional 4 hours at 37°C. After removal of medium, the resulting formazan crystals were dissolved in DMSO (Sigma-Aldrich) for 5 minutes and the plates were read in a spectrophotometer at 540 nm. For suspension cultures, MTS dye (0.37 mg/mL) was added. The cells were incubated for 4 hours and then read spectrophotometrically at 490 nm. Dose response curves were created using GraphPad Prism version 5.01 (GraphPad Software Inc, La Jolla, CA). IC_50_ values were calculated using CalcuSyn (Biosoft, Great Shelford, Cambridge, UK).

### Proteasome subunit quantification

The proteasome constitutive/immunoproteasome subunit enzyme-linked immunosorbent (ProCISE) assay was used to quantitate individual constitutive (β5, β1, and β2) and immunoproteasome (LMP7, LMP2, and MECL1) subunits [29]. Briefly, cell lysate was incubated with a biotinylated proteasome active-site binding probe (PABP). Lysate was then denatured, and subunits bound to PABP were isolated with streptavidin-conjugated sepharose beads. Individual subunits were probed with subunit-specific primary antibodies, followed by HRP-conjugated secondary antibodies. A chemiluminescent substrate was used to generate a luminescent signal associated with bound HRP, which was read on a luminometer. Absolute values of subunit per microgram of total protein were determined based on a purified proteasome standard curve. Statistical significance was determined by Student’s t test.

### Cell viability

Cells were seeded in 6-well plates and incubated overnight. Cultures were left untreated or treated with predetermined IC_50_ doses of CFZ or CDDP and incubated for an additional 48, 72, or 96 hours. After each time point, the media containing any non-adherent cells was collected. Adherent cells were then detached using trypsin, suspended in culture medium, and disaggregated by manual pipetting. After mixing all collected cells with trypan blue (Thermo Scientific), viable cells that excluded the dye and dead cells that stained an intense blue were counted using a hemocytometer.

### Western blot analysis

After various time points, the cells were rinsed with cold phosphate buffered saline (PBS) and harvested in a buffer containing 50 mM Tris HCl (pH 8.0), 150 mM NaCl, 1% Triton X-100, 2 mM EDTA, 5 mM Na_3_VO_4_, 200 μM NaF, 21 μM leupeptin, 230 nM aprotinin, and 1 mM phenylmethylsulfonyl fluoride (PMSF). The cell lysate was sonicated and centrifuged at 10,000 × *g* for 10 min at 4°C. Protein concentration of the resulting supernatant was determined using a 660 nm Protein Assay kit (Thermo Scientific, Rockford, IL). Twenty micrograms of total cell lysate were boiled for 5 min and resolved in a 10% acrylamide/bisacrylamide gel by electrophoresis at 125 V for 105 min. Proteins were then transferred to a polyvinylidene fluoride (PVDF) membrane (Millipore, Billerica, MA). Membranes were blocked with 5% milk in Tris-buffered saline containing 0.1% Tween-20 (TBST) for 30 min before overnight incubation at 4°C with various primary antibodies, typically at a 1:1000 dilution. Blots were rinsed with TBST and incubated for 2 hours at room temperature with secondary antibody at a 1:15,000 dilution. Reactive bands were visualized by exposure to film using Pierce Supersignal West Pico HRP Detection Reagent (Thermo Fisher Scientific, Rockford IL). Blots were stripped in 0.2 M NaOH with shaking for 10 min at room temperature and then re-probed for loading control.

### Drug combination studies

Cells were seeded as described in the proliferation assay section and treated with various concentrations of CFZ and CDDP either simultaneously, CDDP followed by CFZ, or CFZ followed by CDDP. Simultaneously-treated cells were incubated for 96 hours before being analyzed. For CDDP/CFZ, cells were treated with CDDP for 24 hours, followed by CFZ for an additional 72 hours before analysis. For CFZ/CDDP, cells were first treated with CFZ for 48 hours, then with CDDP for an additional 48 hours before analysis. For sequential drug treatments, the first drug remained in the media as the second drug was added. The range of concentrations used for each drug was 4.5 nM to 30 μM. The upper dose (30 μM) is near the estimated blood concentration of CDDP found in patients following administration of the dosage that is used clinically (100 mg/m^2^) [30]. Interactions between CFZ and CDDP were analyzed using the median effect method of Chou and Talalay [31]. In this method, dose–response curves are generated for each agent individually. These results are then used to analyze the results obtained from the combination treatment. A combination index (CI) was generated using CalcuSyn software (Biosoft, Cambridge, United Kingdom) and synergy level classifications were assigned as described in the CalcuSyn manual. A CI of less than 1 indicates synergy (<0.3, strong synergy), a CI of 1 indicates additive effects, and a CI of more than 1 is indicative of antagonistic effects (>3, strong antagonism).

### *In vivo* SHP77 xenograft studies

Studies were performed under approved protocols in accordance with Institutional Animal Care and Utilization Committee guidelines. SHP77 (10^7^) cells were subcutaneously injected into flanks of female *scid* mice. When tumors became palpable (average tumor volume between 200–300 mm^3^), mice were randomized into four treatment groups: 1) Control - vehicle only, 2) CFZ - 3 mg/kg intravenous (IV) injection for two consecutive days for the first week followed by 5 mg/kg IV for two consecutive days repeated weekly until death or sacrifice, 3) CDDP – 4 mg/kg intraperitoneal (IP) injection once per week for 3 weeks, and 4) CFZ – 5 mg/kg IV + CDDP – 4 mg/kg IP. For combination treatment, CFZ (3 mg/kg the first week and 5 mg/kg on subsequent weeks) was given on days 1 and 2 of each week and CDDP was given on day 5 for 3 weeks. Animals were euthanized when tumors reached 2000 mm^3^ or became necrotic. A separate group of mice bearing SHP77 flank xenografts (200–500 mm^3^) were randomized into a vehicle control group or CFZ (5 mg/kg IV) dose group (N = 4) and treated for two consecutive days. 48 hours later, mice were euthanized and tumors were removed and fixed in 10% buffered formalin for pharmacodynamic biomarker analysis.

### Histology and immunohistochemistry

SHP77 flank xenograft tumors were excised and immediately fixed in 10% PBS buffered formalin. Tissues were transferred to 70% ethanol within 24 hours and embedded in paraffin within 3 days. Sectioned tissue sections (0.3 micron slices) were prepared for hematoxylin and eosin (H&E) staining or immunohistochemistry (IHC) from formalin fixed, paraffin embedded (FFPE) blocks. IHC was performed using cleaved caspase-3 antibody (#9661, Cell Signaling, Danvers, MA) diluted 1:400. Tissue sections were stained on a Discovery XT automated system (Ventana Medical Systems, Inc, Tucson, Arizona) using proprietary reagents. Cleaved caspase-3 was detected using the Ventana Anti-Rabbit Secondary Antibody for 16 minutes. Labeling was visualized with the Ventana OmniMap kit followed by counterstaining with hematoxylin. Slides were then dehydrated and coverslips were added as per normal laboratory protocol. Images were acquired using an Olympus BX50 microscope and an Olympus DP72 digital camera with CellSens Digital Image software. One pathologist (Dr. Raymond Nagle, University of Arizona Cancer Center) evaluated each slide and scored the percentage of cells staining positive.

## Results

### CFZ suppresses proliferation in a range of NSCLC and SCLC cell lines

**Figure 1:**
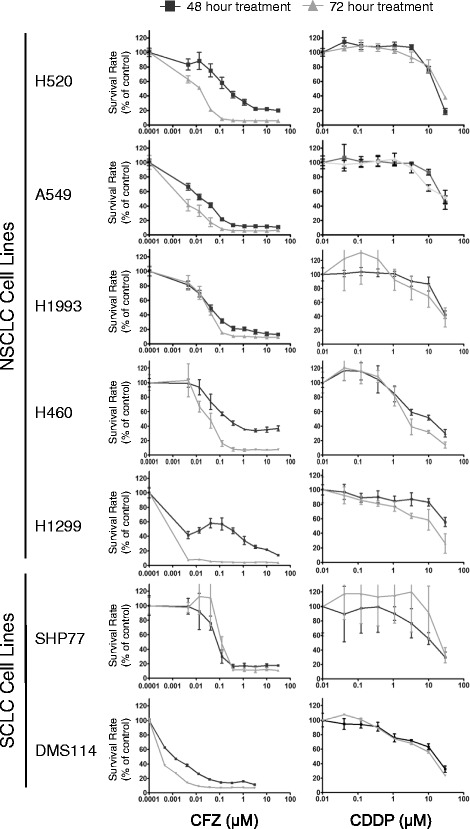
**Dose response curves illustrate the effect of CFZ and CDDP on the growth of lung cancer cell lines.** Cells were exposed to CFZ or CDDP at various concentrations for 48 or 72 hours and assayed by MTT or MTS as described in the Methods section. One representative experiment is shown, n = 6.

A panel of 7 cell lines was used to model the anti-tumor effects of CFZ treatment in lung cancer. We sought to determine the *in vitro* profile of CFZ sensitivity in these cells as compared to CDDP, a platinum-based compound commonly used in lung cancer treatment regimens. MTT or MTS assays were performed on cell lines following 48 or 72 hour treatments. Results from one representative experiment (n = 6) are shown in Figure 1. In each of the cell lines tested, CFZ was more potent than CDDP in inhibiting cell growth. Only slight inhibitory effects were seen at 24 hours, and growth curves at 96 hours were similar to the 72-hour curves (data not shown). IC_50_ values were calculated from the 48, 72, and 96-hour growth curves (Table 1). The IC_50_ values for cell lines treated with CFZ were in the low nanomolar range. In contrast, the IC_50_ values for CDDP treated cells lines were in the micromolar range. CFZ maximal growth inhibition improved over time, reflected by a decreasing IC_50_, for all cell lines with the exception of SHP77 cells, which showed a 60% increase in the IC_50_ value between 48 and 72 hour treatments.

**Table 1:** **Sensitivity of lung cancer cell lines to CFZ and CDDP**

**Cell line**	**CFZ IC**_**50**_ ** (μM)**			**CDDP IC**_**50**_ ** (μM)**		
	**48 h**	**72 h**	**96 h**	**48 h**	**72 h**	**96 h**
**NSCLC**						
H520	0.0769	0.0299	0.0083	19.43	23.24	18.15
A549	0.0901	0.0716	0.0047	25.97	26.53	9.01
H1993	0.0277	0.0172	0.0216	29.60	19.58	15.52
H460	0.1657	0.0631	0.0364	9.02	3.72	2.69
H1299	0.0500	0.0293	<0.0001	>30.0	8.81	3.53
**SCLC**						
SHP77	0.0614	0.0994	0.2031	12.17	21.55	28.57
DMS114	0.0007	0.0002	<0.0001	13.13	8.10	3.47

The determined inhibitory concentrations (IC_50_ values) represent the level of drug that inhibited cell growth by 50%. IC_50_ values (μM) of CFZ at 48 and 72 hours are the mean of two or more independent experiments, n ≥ 12. Other IC_50_ values are the mean of one experiment, n = 6.

### CFZ inhibits proteasome subunits in lung cancer cell lines

**Figure 2:**
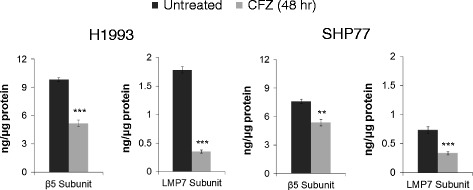
**The c20S subunit β5 and the i20S subunit LMP7 are inhibited in CFZ treated H1993 and SHP77 cells.** Cells left untreated or treated for 48 hours with CFZ (48 hour IC_50_ dosage) were evaluated using the ProCISE assay to determine the level of uninhibited subunits. Mean ± SD are from three pseudo-replicates. Results were statistically significant (***p < 0.001, **p < 0.01) using Student’s t-test.

The ProCISE assay was used to determine which proteasome subunits are inhibited by CFZ in lung cancer cell lines. This immunosorbent assay-based method provides accurate quantitation of proteasome subunits within cell lysate [29]. The abundance of the chymotrypsin-like (CT-L), caspase-like (C-L), and trypsin-like (T-L) subunits of both the constitutive and immunoproteasome was quantified in various NSCLC and SCLC cell lines. Similar to results reported with other cell types [29], CFZ selectively inhibits the CT-L activity of the proteasome in multiple lung cancer cell lines. The activities of the β5 subunit of the constitutive proteasome and the LMP7 subunit of the immunoproteasome were significantly reduced in both the H1993 (NSCLC) and SHP77 (SCLC) cell lines when treated with the predetermined IC_50_ doses of CFZ for 48 hours (Figure 2). Another NSCLC cell line (A549) was tested with similar results (data not shown).

### CFZ reduces cell viability while inducing apoptosis and autophagy

**Figure 3:**
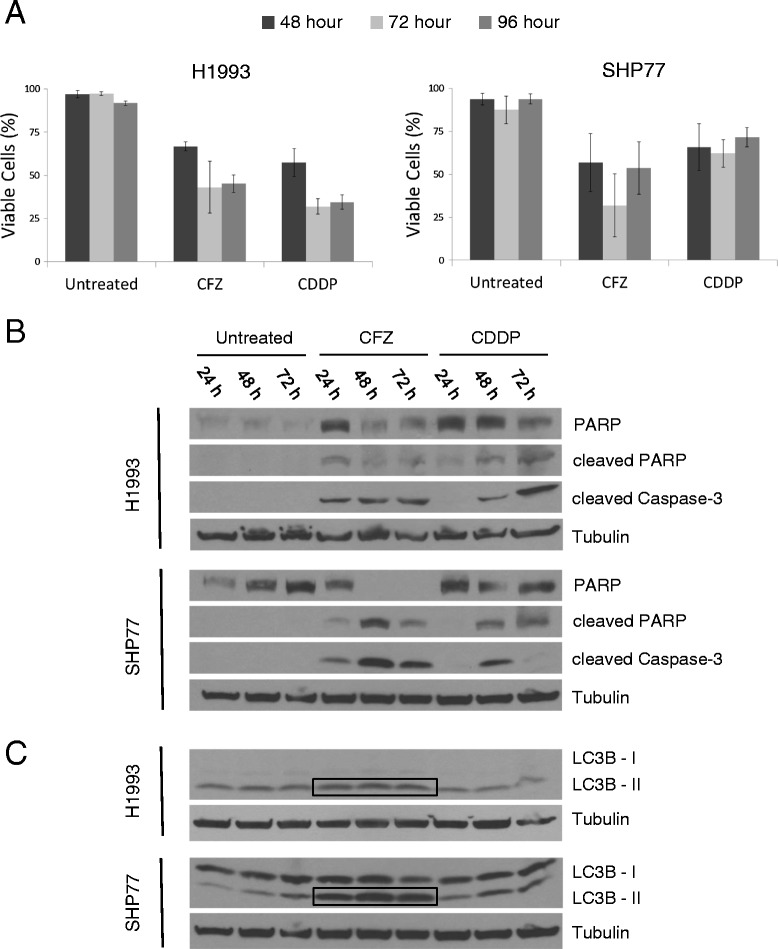
**CFZ treatment reduces cell viability through induction of apoptosis and can induce autophagy.** Cells were left untreated or exposed to CFZ or CDDP at the 48 hour IC_50_ dose. **A)** Viability at 48, 72, and 96 hours was determined by direct cell counting after trypan blue staining. Mean ± SD are shown (n = 4). **B)** Detection of PARP, cleaved PARP, cleaved caspase-3, and **C)** LC3B by immunoblot in total extracts of cells harvested at 24, 48, and 72 hours. Boxed areas highlight the level of LC3B-II in CFZ treated cells which is a marker of autophagy. α-tubulin is shown as a loading control.

To determine the cytotoxic effects of CFZ in lung cancer cell lines, the H1993 NSCLC and SHP77 SCLC cells were treated with predetermined 48 hour IC_50_ doses. Cells were collected at 48, 72, and 96 hour time points and analyzed for viability (Figure 3A). Results demonstrate a strong sensitivity to CFZ *in vitro* with a 40-60% decrease in viable cells. This is similar to CDDP treatment which showed a 30-60% decrease in viability. To determine whether the observed reduction in viability with CFZ treatment occurred via the induction of apoptosis, levels of cleaved PARP and cleaved caspase-3 were evaluated by Western blot. As with CDDP, treatment with CFZ showed induction of these apoptotic markers (Figure 3B).

Some studies suggest that one possible mechanism of resistance to proteasome inhibition is induced autophagic flux [32]. To profile autophagy in our lung cancer cells we examined LC3B which is present in its cytoplasmic form (LC3B-I) or is directly associated with the plasma membrane of autophagosomes (LC3B-II). We noted increased LC3B-II, a marker of autophagy, at 24, 48, and 96 hours in most cell lines tested (Figure 3C and data not shown). A larger increase in LC3B-II was found in the more CFZ-resistant SHP77 cell line as compared to the more CFZ-sensitive H1993 cell line.

### Effects of combining CFZ and CDDP in lung cancer cell lines

Because CDDP is frequently used as part of standard front-line chemotherapy regimens in NSCLC and SCLC, we investigated the combined activity of CFZ and CDDP when added simultaneously for 96 hours on inhibition of cell proliferation using the MTT or MTS assay (Table 2). This combination had mixed results, with antagonism observed in H520 and H1993 cell lines and modest synergy observed in the H460 and SHP77 cell lines. Cell characteristics, such as mutations of EGFR or K-Ras, may alter the effectiveness of first–line platinum-based chemotherapy [33]. To investigate whether sequential administration of agents could improve activity, we treated with CDDP for 24 hours, followed by addition of CFZ for 72 hours, or CFZ for 48 hours followed by addition of CDDP for 48 hours. The first drug remained in the media as the second drug was added. Results were similar regardless of sequence, with antagonism (decrease in efficacy) observed in all cell lines except the H460 cells when CFZ was administered first; in SHP77 cells there was synergy observed regardless of sequencing. Synergy values were not calculated in the H1299 or DMS114 cell lines due to the almost complete inhibition of proliferation observed from combination treatments.

**Table 2:** **Inhibitory activity of CFZ combined with CDDP in lung cancer cell lines**

**Cell line**	**Simultaneous**		**CDDP→CFZ**		**CDDP→CFZ**	
	**IC**_**50**_ ** (nM)**	**Synergy Value**	**IC**_**50**_ ** (nM)**	**Synergy Value**	**IC**_**50**_ ** (nM)**	**Synergy Value**
**NSCLC**						
H520	14.5	Ant.	427.3	Ant. +	87.6	Ant. +
A549	<1	NC	206.2	Ant. +	72.3	Ant. +
H1993	173.1	Ant. +	134.0	Ant. +	200.6	Ant. +
H460	14.8	Syn.	258.3	Ant. +	25.1	Syn.
H1299	<1	NC	<1	NC	<1	NC
**SCLC**						
SHP77	72.8	Syn.	171.8	Syn.	21.7	Syn. +
DMS114	<1	NC	<1	NC	<1	NC

Cells were treated with equivalent doses of CFZ and CDDP either simultaneously, CDDP followed by CFZ, or CFZ followed by CDDP and evaluated 96 hours after initial treatment. IC_50_ values represent the concentration at which the drug combination inhibited cell growth by 50%. Synergy values were determined with Calcusyn software from the combination index (CI) method described by Chou and Talalay [31]. CI interpretation: <0.3 strong synergism (Syn. +), 0.3-0.9 synergism (Syn.), 1–3 antagonism (Ant.), >3 strong antagonism (Ant. +), or not calculated due to high cell death at lowest dose (NC).

### *In vivo* growth inhibition and survival

**Figure 4:**
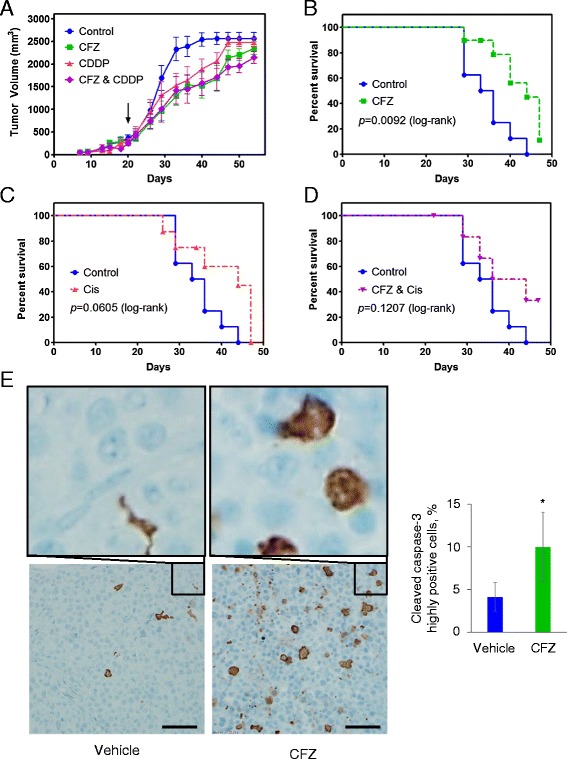
***In vivo*** **responses of SHP77 xenografts to CFZ and CDDP.** Groups of *scid* mice (n = 8) were treated as detailed in the Methods section. **(A)** Mean tumor growth curves of SHP77 xenografts. The x-axis depicts days post tumor cell injection. Drug treatment was initiated on day 20 as indicated by arrow. **(B, C, D)** Kaplan-Meier survival curves of SHP77 tumor bearing mice for control and CFZ treated, control and CDDP treated, and control and CFZ/CDDP combination treated groups. The log-rank (Mantel-Cox) test was performed to evaluate the differences in survival between groups. A significant difference (*p <* 0.05) in survival was found between the control and CFZ treated groups. **(E)** SHP77 xenograft groups (n = 4) were treated two consecutive days with either vehicle or CFZ (5 mg/kg). Tumors were harvested 48 hours after the second dose and fixed sections were processed for immunohistochemical analysis. Representative photographs of immunoreactivity for cleaved caspase-3 at magnification of 60× and 400× are shown. Scale bar on 400× images = 50 μm. The percent of cells with cleaved caspase-3 is significantly higher in the CFZ treated tumors versus the vehicle treated tumors (*p < 0.05 using Student’s t-test).

Groups of *scid* mice (n = 8) were used to evaluate *in vivo* tumor responses to CFZ. Treatment with CFZ monotherapy resulted in tumor growth delay and a significant survival advantage in mice bearing flank SHP77 tumors (Figure 4A,B). Treatment with monotherapy CDDP caused a tumor growth delay, but failed to show a significant survival advantage (Figure 4A,C). Similarly, the combination of CFZ and CDDP demonstrated a tumor growth delay, but no significant survival advantage (Figure 4A, D). It should be noted that the dose of CDDP used was modest in order to minimize toxicity when both drugs were combined. Notably, two mice died within the first week of combination CFZ and CDDP treatment, with deaths being judged to be likely drug-related. Due to the potency observed with CFZ treatment in the DMS114 cell line, we attempted to grow this cell line as flank xenografts in *scid* mice to investigate CFZ *in vivo* efficacy in this model. Unfortunately, we were not able to observe any palpable tumors up to 60 days post-cell inoculation. To determine if CFZ was inducing apoptosis *in vivo,* we analyzed activation and cleavage of caspase-3 in SHP77 xenograft tumors (Figure 4E). A pathologist score showed a significant increase in cleaved caspase-3 in the CFZ treated xenografts versus the vehicle treated xenografts.

## Conclusions

Pre-clinical studies with the first generation reversible proteasome inhibitor BTZ showed that BTZ caused proteasome inhibition that was associated with diminished cell proliferation and increased cell death across a wide variety of NSCLC models [13]. Similarly, we found that CFZ inhibits cell proliferation and induces apoptotic markers in a pathologically and molecularly diverse panel of lung cancer models, including SCLC. IC_50_ values for the cell lines treated with CFZ were all in the low nanomolar range (Table 1). This is similar to the reported BTZ IC_50_ values (30–62.5 nM) found in many of these cell lines [3,34].

*In vitro* testing revealed differences between the SHP77 cells and the other cell lines tested. While the CFZ IC_50_ value decreased over time for all other cell lines tested, the IC_50_ value increased over time for the SHP77 cells, suggesting upregulation of a resistance mechanism or selection of an intrinsically resistant population of cells (Table 2). One explanation for this observation could be the expression of P-glycoprotein (Pgp, MDR1), a drug efflux pump recognized as a major chemotherapy resistance mechanism [35]. Unlike BTZ [36], CFZ has been reported to be a substrate for Pgp [37] and resistance may therefore be mediated by Pgp expression. The SHP77 cells grown *in vitro* express high levels of Pgp as compared to the other cell lines tested (Additional file 1: Figure S1). However, no change in Pgp expression was observed for CFZ treated SHP77 cells over a 96 hour period (data not shown). Therefore the SHP77 resistance mechanism(s) responsible for the increasing IC_50_ value over time for CFZ monotherapy remains unidentified.

We further investigated the efficacy of CFZ using the SHP77 cells in a xenograft model. Because synergy was observed during CFZ and CDDP treatment in the SHP77 cells (Table 2), we investigated both monotherapy and combined activity with CDDP *in vivo*. In spite of Pgp expression in the SHP77 cells, *in vivo* treatment resulted in tumor growth inhibition and a significant survival advantage (Figure 4). This coincided with increased cleaved caspase-3 found in xenograft tumor cells. Unlike in vitro where synergistic activity with CDDP was observed, the combination of CFZ and CDDP *in vivo* did not result in additive growth inhibition or a survival advantage over CFZ monotherapy. It is possible that the *in vivo* tumor microenvironment, which has different levels of growth factors and oxygen supply than cell culture may abrogate cisplatin efficacy and therefore not yield similar results to *in vitro* studies. Clinically, resistance to cisplatin is common in SCLC.

The lack of additive activity with CFZ and CDDP may be due to activation of survival signaling in response to CDDP and/or CFZ. There are multiple mechanisms by which cells acquire resistance to CDDP (reviewed by Galluzzi et al. [38]). Upregulation of heat shock proteins, which has been previously reported in response to proteasome inhibition, is associated with cisplatin resistance in ovarian cancer cells [39]. Autophagy has also been shown to be associated with acquired cisplatin resistance in the A549 NSCLC cell line [40]. We saw increased expression of LC3B-II, a putative marker of autophagy, in response to both CFZ and CDDP treatment *in vitro* in the SHP77 and H1993 cell lines (Figure 3C). This observation suggests that induction of autophagy by CFZ could contribute to the abrogated efficacy of CDDP.

Gaining a greater understanding of resistance mechanisms to proteasome inhibition will be important in further developing CFZ as a lung cancer therapeutic. Mechanisms for both intrinsic and acquired resistance to BTZ have been described [41–43]. In lung cancer cell lines, cross resistance to BTZ and CFZ has been observed in A549 and SW1753 cells, but not in H460 BTZ resistant cells. In these studies, resistance to BTZ was associated with mutant β-subunits within the proteasome core [41]. Resistance to BTZ has also been associated with basal proteasome levels [41]. We did not observe this trend when comparing the basal β5 and LMP7 subunit levels with the corresponding CFZ IC_50_ values of the H1993 and SHP77 cell lines (Figure 1, Table 1) or with the A549 cells (data not shown).

Bcl-2 over-expression in SCLC has been linked with chemotherapy resistance [44]. Bortezomib has been shown to reduce Bcl-2 levels and induce apoptosis in the H526 SCLC cell line [45]. In the A549 spheroid model, Bcl-2 upregulation has been associated with BTZ resistance [46]. In head and neck cancer models CFZ resistance was associated with increased levels of the Bcl-2 family member, Mcl-1 [47]. When CFZ is combined with obatoclax, a pan-BH3 mimetic that inhibits Mcl-1, enhanced apoptosis is observed in diffuse large B-cell lymphoma cell lines [48]. Although treatment with CFZ or CPPD did not significantly modulate Bcl-2 levels in the H1993 or SHP77 cells (Additional file 1: Figure S2), future studies should investigate the combined activity of CFZ with Bcl-2 and/or Mcl-1 inhibitors in lung cancer.

We did not directly compare the anti-proliferative activity of BTZ to CFZ in the current study. However, previous studies suggest that BTZ and CFZ have similar *in vitro* activity in lung cancer cell lines [41]. Because CFZ displays greater specificity and has a different pharmacokinetic profile than BTZ; CFZ may have different activity than BTZ in clinical lung cancer studies [49]. One of the major dose-limiting toxicities of BTZ is peripheral neuropathy (PN). CFZ use is associated with much lower new-onset PN [50], making it a more attractive agent in the clinic. CFZ, unlike BTZ, does not induce neurodegeneration *in vitro*, which may explain its lower rates of PN in clinical trials [24]. CFZ overcomes BTZ resistance, suggesting that selective, irreversible PIs without dose-limiting neurotoxicity may lead to more potent antitumor response and an improved tolerability profile compared with reversible PIs [25].

Single-agent CFZ has been approved in the US for treatment of RRMM based on its efficacy and lack of cumulative toxicities [27]. Our findings reported here support further investigation of CFZ in novel combinations with chemotherapy and targeted agents in SCLC and NSCLC pre-clinical models to help guide the clinical development of CFZ in lung cancer. In addition, further delineation of resistance mechanisms to proteasome inhibition may aid in selection of lung cancer patients for CFZ-based therapy.

## Additional file

Additional file 1: Figure S1. Relative Pgp expression in lung cancer cell lines. Total cell lysate from untreated cells were probed for expression of Pgp. The blot was reprobed for α-tubulin as a loading control. **Figure S2.** Analysis of Bcl-2 expression in H1993 and SHP77 cells. Cells were left untreated or exposed to CFZ or CDDP at the 48 hour IC_50_ dose. Levels of Bcl-2 were determined by immunoblot in total extracts of cells harvested at 24, 48, and 72 hours. α-tubulin is shown as a loading control.

## Abbreviations

Bcl-2: B-cell lymphoma 2
BTZ: Bortezomib
CDDP: Cisplatin or *cis*-diamminedichloroplatinum(II)
CFZ: Carfilzomib
CI: Combination index
I-κB: Inhibitory factor kappa B
LC3B: Microtubule-associated protein-1 light chain-3B
NF-κB: Nuclear factor kappa B
NSCLC: Non-small cell lung cancer
PARP: Poly ADP ribose polymerase
Pgp: P-glycoprotein
PIs: Proteasome inhibitors
PN: Peripheral neuropathy
RRMM: Relapsed and refractory multiple myeloma
SCLC: Small cell lung cancer
