# Supplementary material for: Carfilzomib demonstrates broad anti-tumor activity in pre-clinical non-small cell and small cell lung cancer models
Source: J Exp Clin Cancer Res. 2014 Dec 31;33(1):111. doi: 10.1186/s13046-014-0111-8 (PMC4304157; doi:10.1186/s13046-014-0111-8)
Supplement: Additional file 1: Figure S1. — Relative Pgp expression in lung cancer cell lines. Total cell lysate from untreated cells were probed for expression of Pgp. The blot was reprobed for α-tubulin as a loading control. Figure S2. Analysis of Bcl-2 expression in H1993 and SHP77 cells. Cells were left untreated or exposed to CFZ or CDDP at the 48 hour IC50 dose. Levels of Bcl-2 were determined by immunoblot in total extracts of cells harvested at 24, 48, and 72 hours. α-tubulin is shown as a loading control. [file 13046_2014_111_MOESM1_ESM.pptx]

## Slide 1
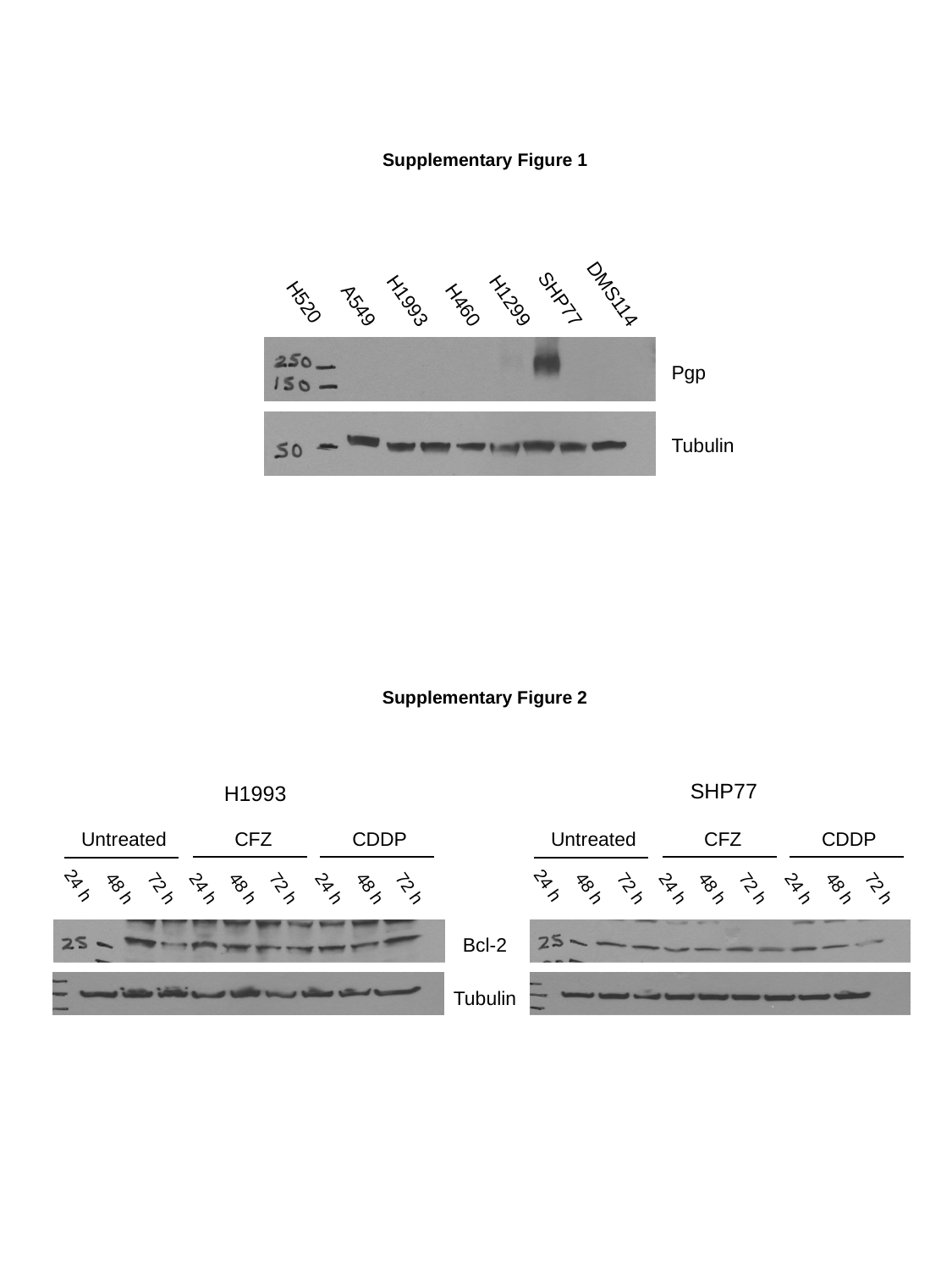

Supplementary Figure 1
DMS114
A549
SHP77
H1993
H520
H1299
H460
Pgp
Tubulin
# Supplementary Figure 2
SHP77
H1993
Untreated
CFZ
CDDP
Untreated
CFZ
CDDP
48 h
48 h
24 h
72 h
48 h
24 h
72 h
48 h
24 h
24 h
72 h
72 h
72 h
48 h
24 h
72 h
48 h
24 h
Bcl-2
Tubulin
